# Three-component synthesis of pyrano[2,3-*d*]-pyrimidine dione derivatives facilitated by sulfonic acid nanoporous silica (SBA-Pr-SO_3_H) and their docking and urease inhibitory activity

**DOI:** 10.1186/2008-2231-21-3

**Published:** 2013-01-05

**Authors:** Ghodsi Mohammadi Ziarani, Sakineh Faramarzi, Shima Asadi, Alireza Badiei, Roya Bazl, Massoud Amanlou

**Affiliations:** 1 Department of Chemistry, Alzahra University, Vanak Square, P.O. Box 19938939973, Tehran, Iran; 2 School of Chemistry, College of Science, University of Tehran, Tehran, Iran; 3 Drug Design and Development Research Center and Department of Medicinal Chemistry, Faculty of Pharmacy, Tehran University of Medical Sciences, Tehran, Iran

**Keywords:** SBA-Pr-SO_3_H, Barbituric acid, Pyrano[2,3-*d*]pyrimidine diones, Multicomponent reaction (MCRs), Urease inhibitory

## Abstract

**Background:**

A straightforward and efficient method for the synthesis of pyrano[2,3-*d*]pyrimidine diones derivatives from the reaction of barbituric acid, malononitrile and various aromatic aldehydes using SBA-Pr-SO_3_H as a nanocatalyst is reported.

**Results:**

Reactions proceed with high efficiency under solvent free conditions. Urease inhibitory activity of pyrano[2,3-*d*]pyrimidine diones derivatives were tested against Jack bean urease using phenol red method. Three compounds of **4a**, **4d** and **4l** were not active in urease inhibition test, but compound **4a** displayed slight urease activation properties. Compounds **4b**, **4k**, **4f**, **4e**, **4j**, **4g** and **4c** with hydrophobic substitutes on phenyl ring, showed good inhibitory activity (19.45-279.14 μM).

**Discussion:**

The compounds with electron donating group and higher hydrophobic interaction with active site of enzyme prevents hydrolysis of substrate. Electron withdrawing groups such as nitro at different position and meta-methoxy reduced urease inhibitory activity. Substitution of both hydrogen of barbituric acid with methyl group will convert inhibitor to activator.

## Introduction

Pyran derivatives are ordinary structural subunits in a variety of important natural products, including carbohydrates, alkaloids, polyether antibiotics, pheromones, and iridoids [1]. Uracil and its fused derivatives, such as pyrano[2,3-*d*pyrimidines, pyrido[2,3-*d*pyrimidines or pyrimido[4,5-*d*pyrimidines are well recognized by synthesis as well as biological chemists.

These annelated uracils have received considerable attention over the past years due to their wide range of biological activity. Compounds with these ring systems have diverse pharmacological properties such as antiallergic [2], antihypertensive [3], cardiotonic [4], bronchiodilator [5], antibronchitic [6], or antitumour activity [7]. The synthesis of the mentioned compounds containing a pyran and an uracil ring poses significant synthetic challenges. Therefore, for the preparation of these complex molecules large efforts have been directed towards the synthetic manipulation of uracils. As a result, a number of reports have described in literature [8-12] which usually require drastic conditions, long reaction times and complex synthetic pathways and the yields are poor. Thus new routes for the synthesis of these molecules have attracted considerable attention in search for a rapid entry to these heterocycles.

The general procedures for the preparation of pyrano[2,3-*d* pyrimidine-2,4(1H,3H)-diones include the reaction of arylidenemalononitriles with barbituric acid under traditional hot reaction conditions [13,14] or microwave irradiation [15]. In these methods the arylidenemalononitriles are previously derived from malononitrile and aldehydes. Recently, direct condensation of aldehydes, malononitrile and barbituric acid in aqueous media has been reported under ultrasound irradiation [16], or catalyzed by diammonium hydrogen phosphate [17].

Different catalysts such as *L*-proline [18], *N*-methylmorpholine [19], [BMIm]BF_4_[20], 1,4-dioxane [13,21], H_14_[NaP_5_W_30_O_110_[22] and [K Al(SO_4_)_2_[23] under heating also 3-deoxy-D-arabino-heptulosonate 7-phosphate (DAHP) [17] and *L*-proline [24] under room temperature condition have been researched for the synthesis of pyrano[2,3-*d*pyrimidine diones derivatives. In addition, Et_3_N was examined under microwave irradiation [25]. The catalyst free procedures for the preparation of the pyrano pyrimidine diones were also investigated using microwave irradiation [15], ultrasonic [16], heating with water [26] and ball-milling technique [27].

Mesoporous materials are a special type of nanomaterials with ordered arrays of uniform nanochannels. These materials have important applications in a wide variety of fields such as separation, catalysis, adsorption, advanced nanomaterials, etc [28-33]. SBA-15 has many advantages such as: largest pore-size mesoporous material with highly ordered hexagonally arranged meso-channels, with thick walls, adjustable pore size from 3 to 30 nm, and high hydrothermal and thermal stability [34-38], therefore it is expected to be an useful catalyst in the synthesis of organic compounds.

The surface of SBA-15 was modified by acidic functional groups (e.g., -SO_3_H) to prepare nano-solid acid catalyst which can use in the synthesis of various heterocyclic compounds [35]. Recently, we have also reported the use of this catalyst for the synthesis of quinoxaline derivatives [39], polyhydroquinolines [40], triazoloquinazolinones and benzimidazoquinazolinones [41].

Moreover, to the best of our knowledge there is no report on the use of these materials as nanoreactors in the synthesis of pyrano pyrimidine diones derivatives. In the present work, we report our results on the research of convenient and green way for the synthesis of pyrano[2,3-*d*]pyrimidine diones derivatives using SBA-Pr-SO_3_H as a nanocatalyst and their urease inhibitory activity was investigated.

## Material and methods

Gc-Mass analysis was performed on a Gc-Mass model: 5973 network mass selective detector, Gc 6890 Agilent. IR spectra were recorded from KBr disk using a FT-IR Bruker Tensor 27 instrument. Melting points were measured by using the capillary tube method with an electro thermal 9200 apparatus. The ^1^H-NMR (250 MHZ) was run on a Bruker DPX, 250 MHZ. Nitrogen adsorption and desorption isotherms were measured at -196°C using a Japan Belsorb II system after the samples were vacuum dried at 150°C overnight. Surface areas were calculated by the Brunauer-Emmett-Teller (BET) method, and pore sizes were calculated by the Barrett-Joyner-Halenda (BJH) method. Thermogravimetry analysis (TGA) was carried out in Perkin Elmer Pyris Diamond instrument from ambient temperature to 800°C using 20°C/min ramp rate.

### Preparation of catalyst

#### Synthesis and functionalization of SBA-15

The nanoporous compound SBA-15 was synthesized and functionalizaed according to our previous report and the modified SBA-15-Pr-SO_3_H was used as nanoporous solid acid catalyst in the following reactions [40-43].

### General procedure for the preparation pyrano[2,3-*d*]pyrimidine diones

**Scheme 1:**
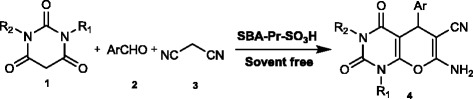
**Three-component synthesis of pyrano[2,3-*d*]pyrimidine dione derivatives.**

The SBA-Pr-SO_3_H (0.02 g) was activated in vacuum at 100°C and then after cooling to room temperature, barbituric acid (0.265 g, 2 mmol), 4-nitrobenzaldehyde (0.362 ml, 2.4 mmol) and malonitrile (0.132 g, 2 mmol) was added to the catalyst in a reaction vessel (Scheme 1). The reaction mixture was heated for 15 min in bath oil at 140°C. After the completion of reaction as indicated by TLC, the generated solid was recrystallized in DMF and ethanol to afford pure product **4**. The resulting solid product was solved in DMF, and then filtered for removing the unsolvable catalyst and then the filtrate was cooled to afford the pure product as a solid. The spectroscopic and analytical data for selected compounds are presented in the following part. The catalyst was washed subsequently with acetonitrile, diluted acid solution, distilled water and then acetone, dried under vacuum and re-used for several times without loss of significant activity.

### Spectral data for product

*7-Amino-6-cyano-5-(3-methylphenyl)-5H-pyrano[2,3-d]pyrimidine-2,4(1H,3H)-diones***4f:** IR (KBr): υ_max_= 3411 and 3320 (NH_2_), 2961 and 2740 (CN), 1733 and 1700 (C=O) cm^-1^. ^1^H NMR (250 MHz, CDCl_3_): δ = 2.47 (s, 3H, CH_3_), 4.13 (s, 1H, CH), 6.95-7.17 (m, 6H, ArH & NH_2_), 11.09 (s, 1H, NH) 12.00 (s, 1H, NH) ppm. Mass (m/z): 296 (M^*+*^), 285, 149 (100).

*7-Amino-6-cyano-5-(3-methoxylphenyl)-5H-pyrano[2,3-d]pyrimidine-2,4(1H,3H)-diones***4h:** IR (KBr): υ_max_= 3183 and 2834 (NH_2_), 2246 and 2200 (CN), 1690 and 1538 (C=O), 1459, 1375 cm^-1^. ^1^H NMR (250 MHz, CDCl_3_): δ = 3.35 (s, 3H, OCH_3_), 4.16 (s, 1H, CH), 6.96-7.78 (m, 4H, ArH), 8.48 (s, 2H, NH_2_) 11.03 (s, 1H, NH), 11.77 (s, 1H, NH) ppm. Mass (m/z): 312 (M^+^), 276, 230, 215.

*7-Amino-6-cyano-5-(2,6-dichlorophenyl)-5H-pyrano[2,3-d]pyrimidinone***4k:** IR (KBr): υ_max_= 3368, 3337, 3148 and 3038 (NH_2_), 2930 and 2196 (CN), 1703 and 1664 (C=O), 1102, 1283, 763 cm^-1^. ^1^H NMR (250 MHz, CDCl_3_): δ = 5.28 (s, 1H, CH), 7.94 (s, 2H, NH_2_), 7.24- 7.36 (m, 5H, -ArH & NH_2_), 11.05 (s, 1H, NH), 12.20 (s, 1H, NH) ppm. Mass (m/z): 352 (M^+^), 316, 282, 267, 257, 191, 167, 122, 82.

*7-Amino-6-cyano-1,3-dimethyl-5-(4-Hydroxyphenyl)-1,5-dihydro-pyrano[2,3-d]pyrimidine-2,4-dione***4l:** IR (KBr): υ_max_= 3415, 3315, 3203 and 3020 (NH_2_), 2192 (CN), 1688, 1659 and 1529 (C=O), 1348 cm^-1^. ^1^H NMR (250 MHz, CDCl_3_): δ = 3.17 (s, 3H, CH_3_), 3.40 (s, 3H, CH_3_), 4.40 (s, 1H, CH), 6.83-8.25 (m, 6H, ArH, & NH_2_) ppm. Mass (m/z): 326 (M^+^), 327(M^+1^), 306, 281, 149 (100).

### Docking approach

AutoDockTools 1.5.4 (ADT) [44], Autogrid 4.2 [45] and Autodock 4.2 [45] were used to prepare input files, calculate grid box and docking experiments. A grid map consisted of 40 × 40 × 40 Å points around the active site was used. The center of the grid was set to the average coordinates of the two Ni^2+^ ions in the α chain of *H. pylori* urease (pdb ID: 3LA4). A Lamarckian genetic algorithm (LGA) was used for the conformational search. The reliability of the applied docking protocol was assessed by re-docking acetohydroxamic acid (AHA) into the active site of the *H. pylori* urease. Each Lamarckian job consisted of 250 runs. The initial population was 150 structures, and the maximum number of energy evaluations and generations was 2.5 × 10^7^. The other parameters were set to default values. The final structures were clustered and ranked according to the most favorable docking energy. This protocol was then similarly applied to all synthesized compounds [46].

### Computational resources

The computational studies were carried out on a computer cluster comprising four sets of HP Prolient ML370-G5 tower servers equipped with two quad-core Intel Xeon E5355 processors (2.66 GHz) and 4 GB of RAM, running a Linux platform (SUSE 10.2).

### Urease inhibitory assay

All the chemicals used were of analytical grade from Merck Co., Germany. All aqueous solutions were prepared in MilliQ (Millipore, USA) water. Jack-bean urease was obtained from Merck (5 units/mg). After proper dilution, the concentration of enzyme solution adjusts at 2 mg/ml which is determined by UV spectroscopy at λ = 280 nm. Urease activity was measured by rapid phenol red urease test contains phenol red 0.1% (w/v) and 100 mM urea in 10 mM phosphate buffer, pH 7.0. Based on this method, the colour change from yellow (pH 6.8) to bright pink (pH 8.2) of phenol red pH indicator as a result of urea hydrolysis to ammonia was measured. The urease activity of the synthesized compounds (10 μl in DMSO) was monitored spectrophotometrically at 560 nm after incubation at 37°C for 30 min [47].

## Results and discussion

In this article, we want to report the use of SBA-Pr-SO_3_H as a nano and green solid acid catalyst and nano- reactor in the synthesis of 7-amino-6-cyano-5-aryl-5H-pyrano[2,3-*d*]pyrimidinones by the Knoevenagel–Michael condensation reaction. The procedure consisted of the mixture of malonitrile, aromatic aldehydes, and barbituric acid derivatives. The reaction proceeded in high yields in the presence of SBA-Pr-SO_3_H as catalyst at room temperature and solvent free conditions to obtain our desired products **4a-4l** (Scheme 1).

First the suitable conditions for the above transformation are examined with various solvents in different temperatures in the presence of SBA-Pr-SO_3_H as nanocatalyst as shown in Table 1. The results revealed when the reaction proceeds in the absence of solvent, the desired product was obtained in high yield (90%) and very short reaction time. By increasing the temperature of the media to 130°C, the reaction time decreases to 15 minutes so the best reaction conditions were obtained (entry 5, Table 1). The same reaction was done without using any catalyst and a very low yield of product was obtained.

**Table 1:** Optimization of the reaction conditions in the synthesis of 4i

**Entry**	**Solvent**	**Time**	**Yield (%)**	**Catalyst**
1	H_2_O	7 h	32	SBA-Pr-SO_3_H
2	EtOH	5 h	31	SBA-Pr-SO_3_H
3	EtOH (1:1)/H_2_O	6 h	28	SBA-Pr-SO_3_H
4	CH_3_CN	5 h	50	SBA-Pr-SO_3_H
5	neat (140°C)	15 min	90	SBA-Pr-SO_3_H
6	neat (140°C)	20 min	30	-

^a^ Reaction conditions: barbituric acid (2 mmol), 4-nitrobenzaldehyde (2.4 mmol), malonitrile (2 mmol) and catalyst (0.02 g).

^b^ Isolated yields.

**Scheme 2:**
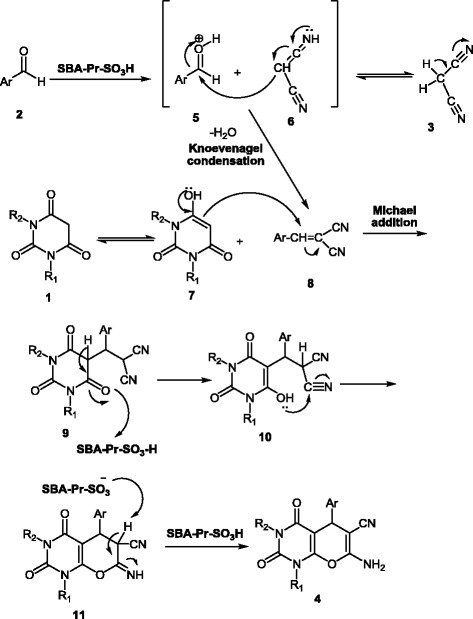
Plausible mechanism for the formation of pyrano[2,3]pyrimidine dione derivatives 4.

A reasonable mechanism for the formation of the product **4** is outlined in Scheme 2. First the oxygen of carbonyl group in benzaldehyde **2** was protonated and malonitrile **3** tautomerized to **6.** The Knoevenagel condensation of compounds **5** and **6** was occurred to form the cyano-olefin **8**. Subsequently, the tautomerized barbituric acid **7** endures nucleophilic attack to **8** and gives the Michael adduct **9**. The intermediate **9** tautomerizes in the presence of acidic catalyst to generate intermediate **10** which cyclizes to give compound **11** which subsequently tautomerized to afford the fully aromatized compound **4**.

Table 2 shows the obtained results in the reaction of a series of representative aldehydes with malononitrile and barbituric derivatives. The most derivatives were obtained in short reaction time ranging 5-45 minutes in high to very high yields. The effect of substituents on the aromatic ring did not show special effects in terms of yields under these reaction conditions.

**Table 2:** **Synthesis of pyrano[2,3-*d*]pyrimidine diones derivatives 4 under optimized conditions**

**Entry**	**Ar**	**R**_**1**_**, R**_**2**_	**Product Structure**^**a**^	**Time (min)**	**Yield**^**b**^**(%)**	**M.p. (°C)**	
						**Found**	**Reported**
1	Ph	Me, Me		5	65	236–238	260–262 [26]
2	2,4-(Cl)_2_-Ph	H, H		20	61	242–243	243–246 [16]
3	4-Me-Ph	H, H		25	62	226–227	225 [18]
4	3-NO_2_-Ph	H, H		15	80	255–226	255–257 [18]
5	4-Cl-Ph	H, H		45	30	234–235	234–237 [15]
6	3-Me-Ph	H, H		30	80	224–225	–
7	4-OMe-Ph	H, H		35	71	280–284	280 [18]
8	3-OMe-Ph	H, H		10	75	200–206	**–**
9	4-NO_2_-Ph	H, H		15	90	227–228	227–229 [15]
10	4-Br-Ph	H, H		15	81	235–236	229–230 [24]
11	2,6-(Cl)_2_-Ph	H, H		15	72	227–228	–
12	4-OH-Ph	Me, Me		20	70	289 (dec)	–

^a^ All the compounds were characterized by IR, NMR, MS, and Mp.

^b^ Isolated yields.

Literature surveys revealed that various conditions have been employed in this reaction as demonstrated in Table 3. The results illustrated that SBA-Pr-SO_3_H was an efficient catalyst in the synthesis of these compounds.

**Table 3:** **Comparison of SBA-Pr-SO_3_H and various catalysts in the synthesis of pyrano[2,3-*d*]pyrimidine diones derivatives 4**

**Entry**	**Catalyst**	**Solvent**	**Condition**	**Time**	**Yield**	**Year/Ref.**
1	Et_3_N	DMF	MW	10–12 min	65–70	2003 [25]
2	*L*-proline	EtOH	Reflux	30 min–12 h	80–92	2010 [18]
3	*N*-methylmorpholine	[bmim][PF_6_]	70°C	15 min	85–89	2004 [19]
4	-	H_2_O	MW	3–5 min	86–94	2004 [15]
5	H_14_[NaP_5_W_30_O_110_]	EtOH	Reflux	30–60 min	85–90	2010 [22]
6	-	1,4-dioxane/H_2_O	80°C	18 min	65–87	1984 [13]
7	[BMIm]BF_4_	[BMIm]BF_4_	90°C	3–5 h	82–95	2005 [20]
8	-	H_2_O	80°C	7.5–11 h	72–81	2007 [26]
9	-	1,4-dioxane/H_2_O	Reflux	1–2 min	60–70	1997 [21]
10	*L*-proline	EtOH	r.t	30–150 min	68–88	2009 [24]
11	-	Ball-milling	r.t	15–90 min	94–99	2009 [27]
12	-	H_2_O	US	1–3 h	62–78	2005 [16]
13	DAHP	EtOH	r.t	2 h	71–81	2008 [17]
14	[KAl(SO_4_)_2_]	H_2_O	80°C	40–50 min	80–90	2010 [23]
15	SBA-Pr-SO_3_H	-	140°C	5–45 min	91	This work

[BMIm]BF_4_: 1-Butyl-3-methylimidazolium Tetrafluoroborate.

DAHP: 3-deoxy-D-arabino-heptulosonate 7-phosphate.

### Preparation of catalyst

**Figure 1:**
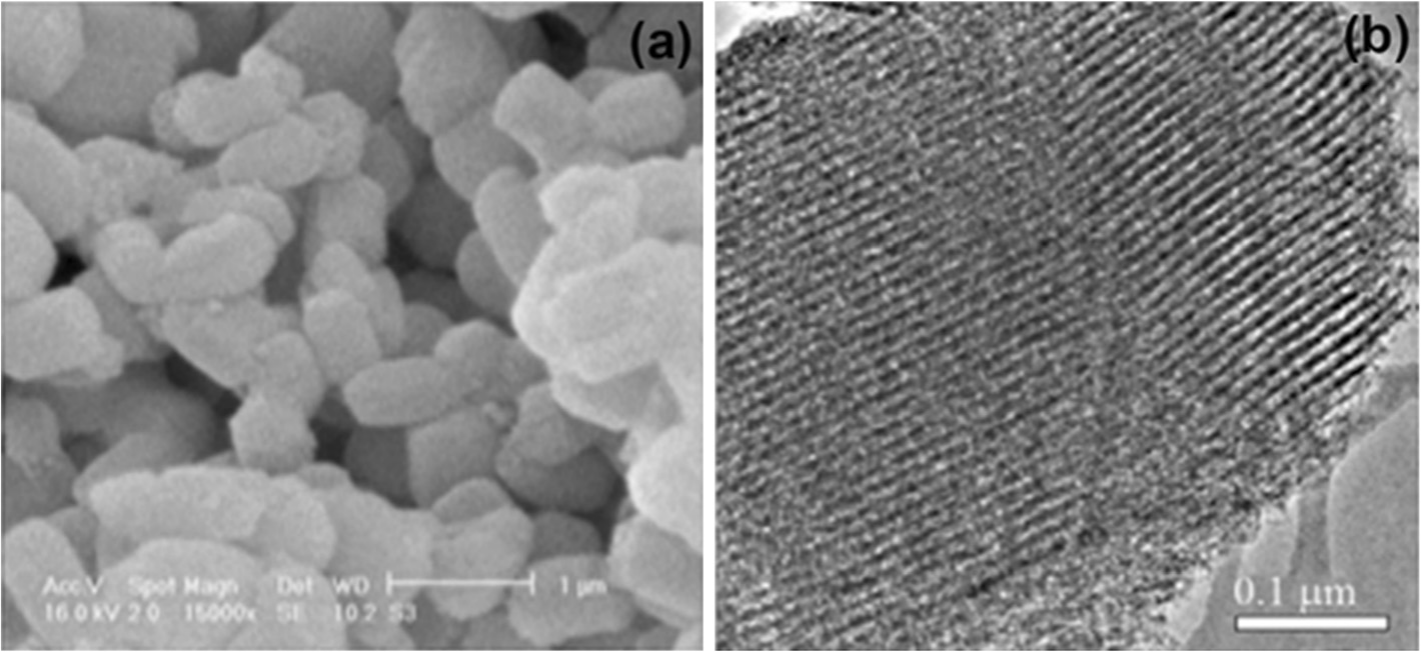
(a) SEM and (b) TEM images of SBA-Pr-SO_3_H.

Pure Nanoporous compound SBA-15 was synthesized according to the well-established method designed by Zhao & coworkers [42] with triblock poly(ethylene oxide)-b-poly(propylene oxide)-b-poly(ethylene oxide) copolymer (Pluronic, EO_20_PO_70_EO_20_, P_123_) as the template. The SBA-15 silica was functionalized with (3-mercaptopropyl)trimethoxysilane (MPTS) and then, the thiol groups were oxidized to sulfonic acid by hydrogen peroxide. Analyzing of the catalyst surface was performed by various methods such as TGA, BET and CHN methods which demonstrated that the propylsulfonic acids were immobilized into the pores. Calculating average pore diameter of the surface area was performed by the BET method and pore volume of SBA-Pr-SO_3_H are 440 m^2^ g^-1^, 6.0 nm and 0.660 cm^3^ g^-1^, respectively, which are smaller than those of SBA-15 due to the immobilization of sulfonosilane groups into the pores [40]. The TGA analysis of SBA-Pr-SO_3_H confirmed the amount of organic groups on SBA-15. The weight reduction of SBA-Pr-SO_3_H in the temperature range between 200-600°C indicated that the amount of organic group was 1.2 mmol/g. SEM image of SBA-Pr-SO_3_H (Figure 1a) shows uniform particles about 1μm. The same morphology was observed for SBA-15. It can be concluded that morphology of acid catalyst was saved without change during the surface modifications. On the other hand, the TEM image (Figure 1b) reveals the parallel channels, which resemble to the pores configuration of SBA-15. This indicates that the pore of SBA-Pr-SO_3_H was not collapsed during two steps reactions.

The pyrano[2,3-*d*pyrimidine diones structurally similar to barbituric acids. The antibacterial and urease inhibitory activity of barbituric acid derivatives were reported [46,48,49]. Many urease inhibitors have been synthesized and tested, but because of their toxicity and instability use of them in vivo is impossible [48-50]. Thus, the search is still on for finding strong and specific urease inhibitors.

As shown in Table 4, all prepared pyrano[2,3-*d*]pyrimidine diones are demonstrated different profile of activity. This might be due to similarity of synthesised compounds to substrate of enzyme. While compounds **4a**, **4d** and **4l** were not active in urease inhibition test, compound **4a** displayed slight urease activation properties. Compounds **4b**, **4k**, **4f**, **4e**, **4j**, **4g** and **4c** with hydrophobic substitutes on phenyl ring, show good inhibitory activity (Table 4). These compounds with electron donating group and subsequent hydrophobic interaction with active site of enzyme prevents the hydrolysis of substrate. Electron withdrawing groups such as nitro, 3-methoxy reduced urease inhibitory activity due to decreasing partial charge on nitrogen atoms of barbiturate moiety on pyrano[2,3-*d*]pyrimidine ring which is essential for inhibitory activity. Substitution of both hydrogen of barbituric acid with methyl groups will convert the inhibitor to activator.

**Table 4:** **Urease inhibitory activities (IC_50_ in μM) and interaction energies (kcal mol^−1^) of pyrano[2,3-*d*]pyrimidine diones derivatives 4**

**Entry**	**Product**	**Docking energy (kcal/mol)**	**IC**_**50**_**(μM)**	**Structure**
1	**4a**	−5.46	98.77	
2	**4b**	−6.43	19.45	
3	**4c**	−5.65	71.92	
4	**4d**	−5.46	98.93	
5	**4e**	−5.87	49.65	
6	**4f**	−5.98	41.13	
7	**4g**	−5.73	62.92	
8	**4h**	−5.42	106.29	
9	**4i**	−4.85	279.14	
10	**4j**	−5.85	51.31	
11	**4k**	−5.99	41.0	
12	**4l**	−5.41	107.62	
13		−3.98	21.0	Thiourea

^a^ Green and red arrows are presented H-bond donor and acceptor interactions, respectively.

^b^ Yellow areas are parts with hydrophobic interactions.

^c^ Blue lines depict the electrostatic interactions.

^*^ The IC_50_ values for activator.

## Conclusions

**Figure 2:**
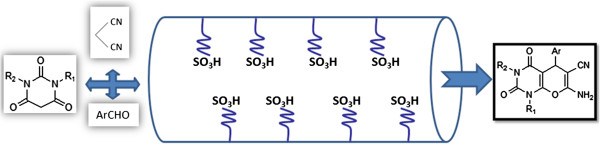
SBA-Pr-SO_3_H act as a nano-reactor in this reaction.

In conclusion we have developed a nano-catalyzed multicomponent synthesis of pyrano pyrimidine diones in good to very good yields. In comparison with previous investigations (Table 3), we presented SBA-Pr-SO_3_H as an efficient and active nano-reactor (Figure 2). Our method is simple as no special apparatus, reagents or chemicals, and work up are required, and the formed compound is filtered and purified just by simple crystallization. This synthesis is also advantageous in terms of atom economy as well as is devoid of any hazardous chemicals. The urease inhibitory activity of pyrano[2,3-*d*]pyrimidine dione derivatives were reported for first time.

## Competing interest

The authors declare that they have no competing of interests related to this publication.

## Authors’ contributions

GMZ: Collaboration in design and identifying of the structures of target compounds, manuscript preparation. SF: Synthesis of the intermediates and some target compounds. SA: Synthesis of some target compounds and collaboration in identifying of the structures of target compounds. AB: Synthesis and characterization of nano catalyst. RB: Collaboration in docking study and evaluation of urease inhibitory test. MA: Collaboration in design of docking study, management of urease test and manuscript preparation. All authors read and approved the final manuscript.
